# A pharmacoepidemiological nested case-control study of risk factors for venous thromboembolism with the focus on diabetes, cancer, socioeconomic group, medications, and comorbidities

**DOI:** 10.1177/14791641241236894

**Published:** 2024-06-21

**Authors:** Lasse Myllylahti, Leo Niskanen, Riitta Lassila, Jari Haukka

**Affiliations:** 1 Department of Hematology, Comprehensive Cancer Center, Helsinki University Hospital, Helsinki, Finland; 2 Department of Internal Medicine, 60667 Päijät-Häme Central Hospital, Lahti, Finland; 3 Institute of Biomedicine, University of Eastern Finland, Kuopio, Finland; 4 Unit of Coagulation Disorders, Department of Hematology, Comprehensive Cancer Center, Helsinki University Hospital, Helsinki, Finland; 5 Research Program Unit in Systems Oncology, 3835 University of Helsinki, Helsinki, Finland; 6 The Finnish Institute of Health and Welfare, Helsinki, Finland; 7 Department of Public Health, Clinicum, Faculty of Medicine, 3835 University of Helsinki, Helsinki, Finland

**Keywords:** Venous thromboembolism, pulmonary embolism, deep vein thrombosis, diabetes, cancer-associated thrombosis, prothrombotic drugs

## Abstract

**Objectives:**

A pharmacoepidemiological study to assess VTE risk factors in a diabetes-rich population.

**Methods:**

The study comprised 299,590 individuals. We observed 3450 VTEs and matched them with 15,875 controls using a nested case-control approach and collected data on comorbidities and prescriptions. By multivariable conditional logistic regression, we calculated ORs with 95%CIs for comorbidities and medications to evaluate their associations with VTE.

**Results:**

Diabetes (aOR 2.16; 95%CI 1.99–2.34), inflammatory bowel disease (1.84; 1.27–2.66), and severe psychiatric disorders (1.72; 1.43–2.05) had the strongest associations among the non-cancer comorbidities. Pancreatic (12.32; 7.11–21.36), stomach (8.57; 4.07–18.03), lung and bronchus (6.26; 4.16–9.43), and ovarian (6.72; 2.95–15.10) cancers were ranked as high-risk for VTE. Corticosteroids, gabapentinoids, psychotropic drugs, risedronic acid, and pramipexole were most strongly associated (aOR exceeding 1.5) with VTE. Insulin (3.86; 3.33–4.47) and sulphonylureas (2.62; 2.18–3.16) had stronger associations than metformin (1.65; 1.49–1.83). Statins and lercanidipine (0.78; 0.62–0.98) were associated with a lowered risk of VTE.

**Conclusions:**

In this cohort, with 50% diabetes prevalence, pancreatic, stomach, lung and bronchus, and ovarian cancers were strongly associated with VTE. Corticosteroids, gabapentinoids, and psychotropic medications had the strongest associations with VTE among medications. This may be valuable for generating hypotheses for the further research. Lercanidipine may be a novel protective medication against VTE.

## Introduction

Venous thromboembolism (VTE), with its typical manifestations of deep vein thrombosis (DVT) and pulmonary embolism (PE), is a major global health issue, with an incidence of 1–2 per 1000 person-years.^1–5^ Over time, the understanding of VTE has developed from a complication of major surgery or terminal illness to a multifactorial disease with several associated inherited, acquired, environmental, and social risk factors.^1–5^ VTE is a highly heterogeneous disease, ranging from asymptomatic radiological co-incidence to immediately life-threatening acute massive PE with up to a 10% acute-phase direct thrombosis-related case mortality.^6^ Accompanied by common post-thrombotic consequences that compromise either the acute-phase survivors´ quality of life^7,8^ or long-term prognosis,^9^ the burden of VTE at the population level is substantial. Despite all landmark epidemiological research,^1–5^ predicting who will develop VTE remains challenging, as some people with a notable risk factor burden will never develop VTE, while many cases (25–50% of first VTE incidents) are idiopathic and have no identifiable risk factors.^4^

Regarding non-cancer comorbidities, many conditions are associated with increased VTE risk, including inflammatory bowel disease, chronic heart failure, neurologic paralytic conditions, autoimmune disorders like systemic lupus erythematosus or systemic vasculitis, hyperthyroidism, or chronic kidney disease.^1–5^ Diabetes mellitus with its complications is an emerging and burdensome public health problem.^10^ Atherosclerosis and diabetes have a well-established connection, and prothrombotic alterations in coagulation cascade and fibrinolytic function have been reported in diabetes.^11,12^ VTE is not infrequent among diabetes patients, but it is not proven if diabetes by itself is an independent risk factor for VTE^13,14^ after adjustment for diabetes-related confounders and co-morbidities. Traditional general VTE risk factors like major surgery or hospitalization also predispose diabetes patients to thrombosis,^15^ and additionally, type 1 diabetes^16^ may carry stronger VTE risk compared to type 2 diabetes,^16^ and female patients with diabetes may be more prone to VTE than male diabetes patients.^17^ Impact of the glycemic control with the risk of VTE is not clear^18^ whereas occurrence of hypoglycemia seems to predispose to long-lasting prothrombotic state.^19^ Of the common acquired risk factors of VTE, cancer makes a significant contribution, and cancer-associated thrombosis has attracted extensive scientific interest in recent years.^20^ The risk of VTE varies considerably depending on the tumor type, and is the highest for pancreatic, brain, lung, and hematologic cancers, whereas breast and prostate cancers generally have the lowest thrombotic potential.^20^ Among cancer-free diabetes patients with first-time episode of VTE, incidence of gallbladder and biliary tract cancer, pancreas cancer, and ovarian cancer were highest in 1-year study period.^21^

Certain medications are associated with an increased risk of VTE incidence, notoriously estrogen-containing oral agents.^22,23^ Glucocorticoids,^22,24^ and many oncological medications^22,25,26^ are also prothrombotic. Additionally, exposure to antipsychotics or antidepressants^22,27^ are associated with VTE, although the causation and specific mechanisms remain uncertain. Beyond actual antithrombotic drugs, statins have been studied as VTE-protecting agents with promising,^28,29^ but also insignificant^30^ results. With respect to diabetes medications, metformin may have a protective effect against VTE^31^ while SGLT-2 and DDP4 inhibitors seems to be neutral.^32,33^ It is noteworthy that these drugs do not predispose to hypoglycemia.

We conducted this nested case-control study to assess potential VTE risk factors and prothrombotic or protective medications in specific diabetes-rich population with an average age of 60. As our data source we used previously described FIN-CARING2 retrospective cohort^34^ consisting of almost 400,000 individuals, of which 50% had diabetes and circa 3400 developed VTE in 16 years of follow-up.

## Methods

### Study design and population

**Figure 1.**
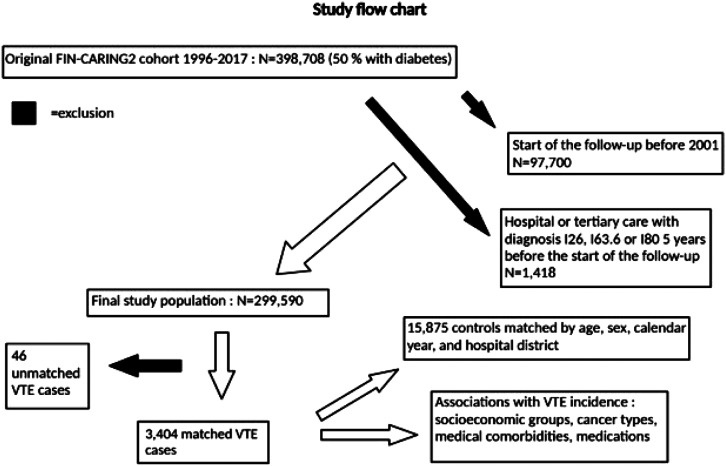
Flow chart of the study. Original cohort, exclusions, final study population, cases, and controls are presented.

The original study population of FIN-CARING2 was based on the multinational CARING project. FIN-CARING2 focused on cancer risk and insulin analogues in Finland and consisted of 398,708 individuals (50% with diabetes), as earlier described by Niskanen et al.^34^ (Figure 1). The beginning of the follow-up was the date of the first diabetes medication prescription and the study period of the original cohort was 1996–2017. In our study, we excluded people with the start of the follow-up before 2001 (*N* = 97,700), leading to 16 years of follow-up. Exclusion was carried out in order to guarantee at least 5-year history of records before the start of the follow-up. People who had received hospital or tertiary care during the 5 years before the start of follow-up with ICD-codes I26 Pulmonary embolism, I63.6 Cerebral infarction due to cerebral vein thrombosis, non-pyogenic, and I80 Diseases of veins, lymphatic vessels, and lymph nodes, not elsewhere classified (*N* = 1418) were also excluded to rule out recurrent VTE events. People with recurrent VTE are a special high-risk group, and their inclusion could have confounded the associations of our study.^1–5^ Thus, the final study population consisted of *N* = 299,590 individuals. The definition of VTE in our study included any of the diagnosis I26, I63.6 and I80. Our national real-world practice is to use the ICD-codes I80 for DVT, and I26 for PE, rather than I82, which captures acute embolism and thrombosis of other specified or unspecified veins. In addition, our previous publication used the same diagnosis codes.^23^ Our study was accepted by the Helsinki University Ethics Committee (REF 02/2012).

### Nested case-control design

We observed 3450 (1.15% of study population) VTE cases and sampled them using the nested case-control approach aiming five controls for each case, matched by age (accuracy of 1 year), sex, hospital district, and the year of the start of the follow-up. Forty-six cases could not be matched because data did not include any potential individuals with correct matching criteria, resulting in one control for 124 cases, two for 61 cases, three for 144 cases, four for 112 cases, and five controls for 2930 cases. Thus, the number of included VTE cases was 3404, and the total number of controls was 15,875. We acquired the baseline data on age, sex, socioeconomic group, cancer diagnosis and type (10-year period before VTE incidence). The data of prevalent comorbidities were based on special reimbursement rights, and the following comorbidities were included: hypothyroidism (reimbursement code 104 including ICD-10 C73, E03, E89.0), severe psychiatric disorders (reimbursement code 112 including ICD-10 F01, F03, F06.0-F06.3, F20–F25, F28, F29, F30.1, F30.2, F31, F32.3, F33.3, F84, G10, G20, G30.0, G30.1, G30.8, G30.9, G31.0, G35, G40.9), chronic heart failure (reimbursement code 201 including ICD-10 I11.0, I13, I50, I97.1, P29.0), coronary artery disease (reimbursement code 206 including ICD-10 I20–I22, I24.0, I25), hypertension (reimbursement code 205 including ICD-10 I10–I13, I15, I27.0, I27.2), chronic arrhythmias (reimbursement code 207 including ICD-10 I47–I49), and inflammatory bowel disease (reimbursement code 208 including ICD-10 K50, K51) (Table 3). The data were obtained from registries of The Social Insurance Institution of Finland (SII) (permission Kela 16/522/2012, providing medications and reimbursement rights), Statistics Finland (permission TK-53-214-12) and the Finnish Cancer Registry (THL/264/5.05.00/2012). There was no missing data in used variables.

### Medications and predefined exposures

We also collected data on the ambulatory use of drugs by anatomical therapeutic chemical (ATC) classification A08A, A10, C, G03, H01-03, L01-04, M05, N03-07, using the SII registry (permission KELA 16/522/2012) as the data source (Supplemental Table 1). A specific drug was included in the analyses if there were at least 50 cases and controls in both the exposed and unexposed groups. To be classed as exposed, an individual had to have redeemed at least three prescriptions in the year before the index date. We also applied another approach to variable selection. Among the available prescription data, we rated certain predefined agents as high-risk medication for VTE and certain agents as protective, based on the previous literature,^21–29^ and analyzed the associations with VTE incidence of these predefined drugs (Supplemental Table 2).

### Statistical modeling

We analyzed the data using multivariable conditional logistic regression, which takes matching into account. Each drug exposure was analyzed separately with and without adjustment for the background variables: socioeconomic group, cancer diagnosis and type; prevalence of comorbidities, including diabetes, hypothyroidism, severe psychiatric disorders, chronic heart failure, coronary artery disease, hypertension, chronic arrhythmias, and inflammatory bowel disease. These variables we added into models, as potential confounders that may have an effect both on exposure and outcome. The results were reported as odds ratios (OR) with 95% confidence intervals (CI). The data were analyzed by R (R Core Team. R: A Language and Environment for Statistical Computing. Vienna, Austria: R Foundation for Statistical Computing, 2021 https://www.R-project.org/). BioRender.com® was used for Figure 1.

## Results

### Baseline characteristics

The mean age of our study population was 66, and 50% were female (Table 1). Regarding comorbidities, diabetes, severe psychiatric disorders, chronic heart failure, hypertension, and coronary artery disease were more prevalent in the case group. Cancer was diagnosed in 18.4% of cases (10-year period before VTE incidence), whereas the cancer prevalence in the control group was 8.5% (Table 2). Because of the nature of the original cohort, the total prevalence of diabetes was high, 50%.

**Table 1.** Basic characteristics of study population.

		Controls	Cases	Overall
*n*		15,875	3404	19,279
Mean age (SD)		65.9 (12.2)	66.3 (12.7)	66.0 (12.3)
Sex (female, %)		7954 (50.1)	1715 (50.4)	9669 (50.2)
Diabetes (yes, %)		7338 (46.2)	2322 (68.2)	9660 (50.1)
Socioeconomic group (%)	Upper-level employees	920 (5.8)	109 (3.2)	1029 (5.3)
	Self-employed	728 (4.6)	119 (3.5)	847 (4.4)
	Lower-level employees	1363 (8.6)	218 (6.4)	1581 (8.2)
	Manual workers	1584 (10.0)	299 (8.8)	1883 (9.8)
	Students	83 (0.5)	26 (0.8)	109 (0.6)
	Pensioners	10177 (64.1)	2384 (70.0)	12561 (65.2)
	Others	1020 (6.4)	249 (7.3)	1269 (6.6)
Thyroid insufficiency (yes, %)		638 (4.0)	171 (5.0)	809 (4.2)
Severe psychotic and other severe mental disorders (yes, %)		508 (3.2)	215 (6.3)	723 (3.8)
Chronic heart failure (yes, %)		619 (3.9)	221 (6.5)	840 (4.4)
Chronic hypertension (yes, %)		5194 (32.7)	1330 (39.1)	6524 (33.8)
Coronary artery disease (yes, %)		2405 (15.1)	729 (21.4)	3134 (16.3)
Chronic arrhythmias (yes, %)		635 (4.0)	143 (4.2)	778 (4.0)
Ulcerative colitis and crohn’s disease (yes, %)		113 (0.7)	45 (1.3)	158 (0.8)

**Table 2.** Cancer prevalence.

		Controls (%)	Cases (%)	Overall (%)
*n*		15,875	3404	19,279
Cancer^a^		1350 (8.5)	628 (18.4)	1978 (10.3)
No cancer		14525 (91.5)	2776 (81.6)	17301 (89.7)
Cancer type	Prostate cancer	401 (2.5)	95 (2.8)	496 (2.6)
	Breast cancer	209 (1.3)	53 (1.6)	262 (1.4)
	Other cancers	129 (0.8)	91 (2.7)	220 (1.1)
	Skin cancer	148 (0.9)	44 (1.3)	192 (1.0)
	Colon cancer	96 (0.6)	43 (1.3)	139 (0.7)
	Bronchus and lung cancer	49 (0.3)	55 (1.6)	104 (0.5)
	Cancer of hematopoietic and reticuloendothelial system	55 (0.3)	44 (1.3)	99 (0.5)
	Cancer of corpus uteri	52 (0.3)	29 (0.9)	81 (0.4)
	Bladder cancer	55 (0.3)	23 (0.7)	78 (0.4)
	Pancreatic cancer	19 (0.1)	50 (1.5)	69 (0.4)
	Rectum cancer	41 (0.3)	22 (0.6)	63 (0.3)
	Kidney cancer	42 (0.3)	20 (0.6)	62 (0.3)
	Lymphoma	29 (0.2)	25 (0.7)	54 (0.3)
	Stomach cancer	14 (0.1)	19 (0.6)	33 (0.2)
	Ovarian cancer	11 (0.1)	15 (0.4)	26 (0.1)

^a^10-year period before incident VTE was diagnosed.

### Socioeconomic group, medical comorbidities, cancer, and associations with VTE

**Figure 2.**
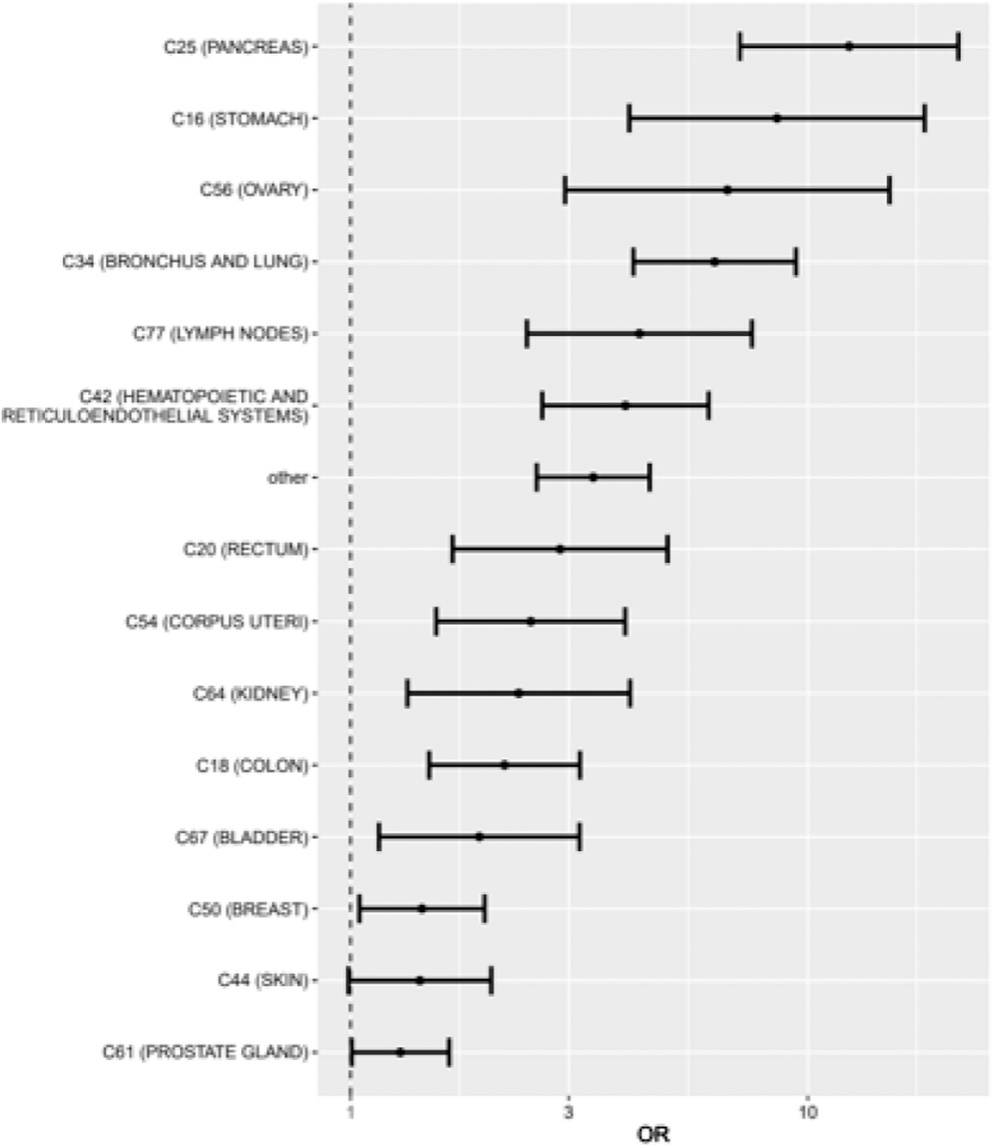
Cancer types and their associations with VTE incidence. Adjusted odds ratios with 95% confidence intervals. Pancreatic, stomach, ovarian, bronchus, and lung cancers have the strongest associations.

By conditional logistic regression analysis, we calculated adjusted ORs (aORs) with 95% CI for incidence of VTE among socioeconomic groups and medical comorbidities, including cancers (Table 3, Figure 2). We adjusted for all background variables in these analyses. Students (aOR 2.38; CI 1.36–4.19) and pensioners (2.31; 1.81–2.94) had the strongest associations with VTE among the socioeconomic groups. The strongest associations among non-cancer comorbidities were with diabetes (2.16; 1.99–2.34), inflammatory bowel disease (1.84; 1.27–2.66), and severe psychiatric disorder (1.72; 1.43–2.05). With respect to the cancer types, pancreatic (12.32; 7.11–21.36), stomach (8.57; 4.07–18.03), ovarian (6.72; 2.95–15.10), and bronchus and lung (6.26; 4.16–9.43) cancers had the highest aORs for VTE incidence (Figure 1). In contrast, breast, skin, and prostate cancers were associated with lower risk compared to other cancer types.

**Table 3.** Associations of socioeconomic groups and comorbidities with VTE.

		aOR^a^ (95% CI^b^)
Socioeconomic group	Self-employed	1.39 (1.04–1.86)
	Lower-level employees	1.35 (1.05–1.74)
	Manual workers	1.49 (1.16–1.90)
	Students	2.38 (1.36–4.19)
	Pensioners	2.31 (1.81–2.94)
	Others	1.92 (1.49–2.47)
Diabetes (yes vs no)		2.16 (1.99–2.34)
Hypothyroidism (yes vs no)		1.11 (0.92–1.33)
Severe psychotic and other severe mental disorders (yes vs no)		1.72 (1.43–2.05)
Chronic heart failure (yes vs no)		1.38 (1.15–1.65)
Chronic hypertension (yes vs no)		1.10 (1.01–1.19)
Coronary artery disease (yes vs no)		1.41 (1.27–1.56)
Chronic arrhythmias (yes vs no)		0.83 (0.68–1.02)
Ulcerative colitis and crohn’s disease (yes vs no)		1.84 (1.27–2.66)

^a^Odds ratio adjusted for all background variables (cancer diagnosis and type; prevalence of comorbidities, including diabetes, hypothyroidism, severe psychiatric disorders, chronic heart failure, coronary artery disease, hypertension, chronic arrhythmias, and inflammatory bowel disease).

^b^Confidence interval.

### Diabetes medications and VTE

We calculated crude ORs and ORs adjusted for the socioeconomic group for different diabetes medication combinations (Table 4). Insulin-containing regimens had the strongest associations with VTE (insulin aOR 3.86; 3.33–4.47, insulin + sulphonylurea aOR 3.55; 1.91–6.58, insulin + sulphonylurea + metformin 3.59; 1.85–6.97) (Table 4). Diabetes without medication had an aOR of 2.60 (2.34–2.90) while metformin monotherapy had the lowest aOR (1.65; 1.49–1.83) among diabetes medication regimens.

**Table 4.** Diabetes medications and associations with VTE.

	Controls (%)	Cases (%)	Overall	OR^a^ (95%CI^b^)	aOR^c^ (95%CI)
*n*	15,875	3404	19,279		
No diabetes	8537 (53.8)	1082 (31.8)	9619 (49.9)	1.00 (1.00–1.00)	1.00 (1.00–1.00)
Diabetes, no medication	2127 (13.4)	762 (22.4)	2889 (14.6)	2.67 (2.40–2.96)	2.61 (2.34–2.90)
Insulin	669 (4.2)	368 (10.1)	1037 (5.4)	3.98 (3.44–4.60)	3.86 (3.33–4.47)
Sulphonylurea	509 (3.2)	195 (5.7)	704 (3.7)	2.71 (2.25–3.26)	2.62 (2.18–3.16)
Insulin+sulphonylurea	31 (0.2)	18 (0.5)	49 (0.3)	3.80 (2.15–7.38)	3.55 (1.91–6.58)
Metformin	3004 (18.9)	678 (19.9)	3682 (19.0)	1.68 (1.51–1.87)	1.65 (1.49–1.83)
Insulin+metformin	532 (3.4)	179 (5.3)	711 (3.7)	2.55 (2.12–3.07)	2.48 (2.06–2.99)
Sulphonylurea+metformin	436 (2.7)	109 (3.2)	545 (2.8)	1.91 (1.53–2.39)	1.86 (1.48–2.33)
Insulin+ sulphonylurea +metformin	30 (0.2)	13 (0.4)	43 (0.2)	3.49 (1.80–6.74)	3.59 (1.85–6.97)

^a^Odds ratio.

^b^95% confidence interval.

^c^Odds ratio adjusted for socioeconomic group.

### Medications and associations with VTE

**Figure 3.**
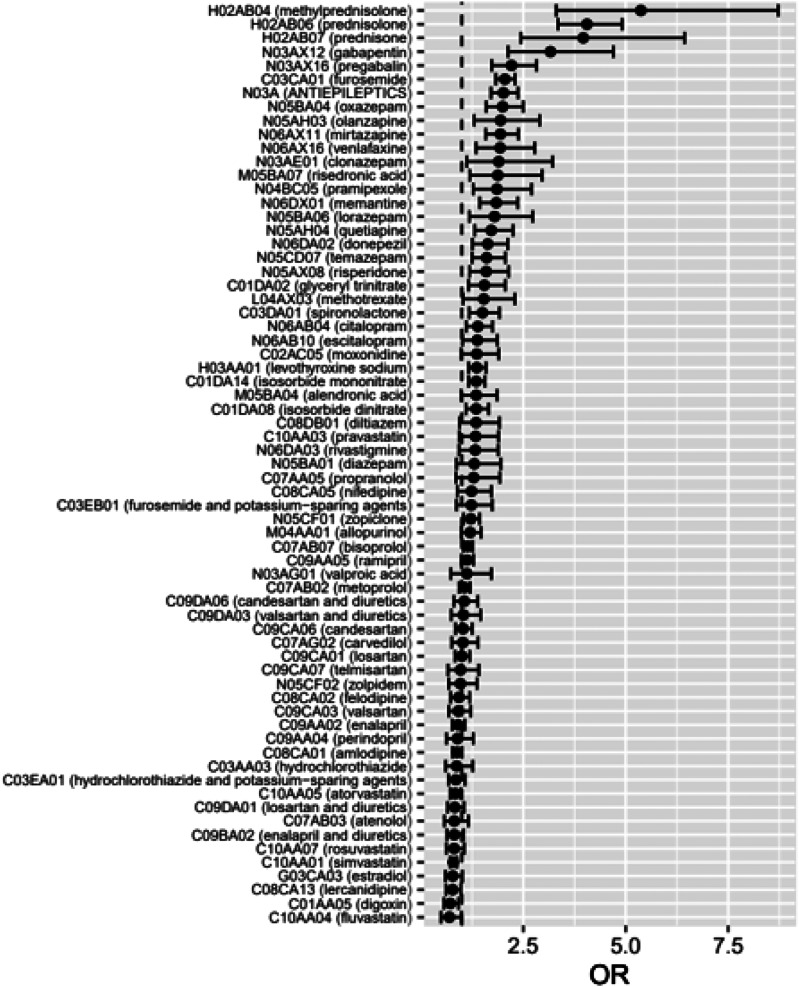
Medications and their associations with VTE incidence. Adjusted odds ratios with 95% confidence intervals are shown. Corticosteroids and gabapentinoids show the strongest associations.

**Figure 4.**
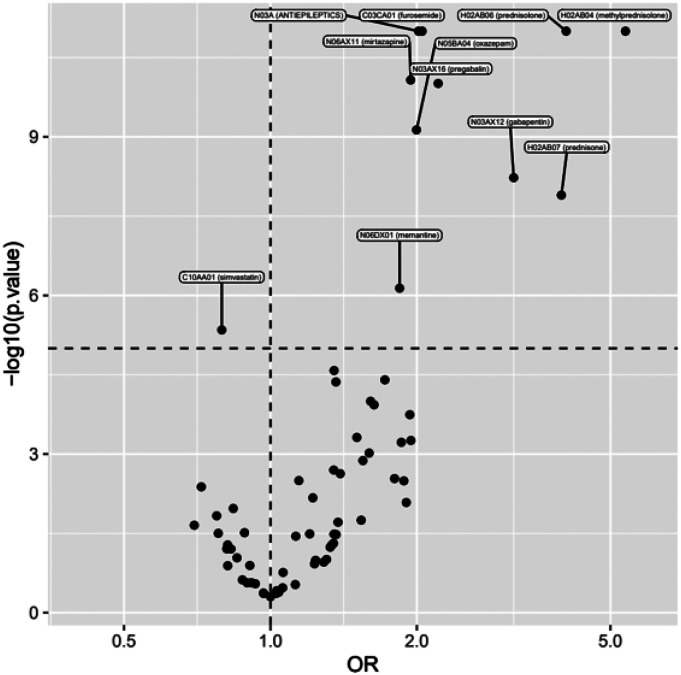
Medications and their associations with VTE incidence. A volcano plot highlights the strongest and statistically most significant associations. Adjusted odds ratio is given on *X*-axis, −log10 of *p*-value on *Y*-axis.

After analyzing diabetes medications, we calculated crude ORs and ORs adjusted for all background variables for other ambulatory medications (Table 5). The detailed data on drug exposures and the associations with VTE incidence reveal a strong association between oral corticosteroids (methylprednisolone (aOR 5.37; 95% CI 3.30–8.73), prednisolone (4.06; 3.35–4.91) and prednisone (3.96; 2.44–6.44)) and VTE incidence (Figures 3 and 4). Gabapentin, an antiepileptic and widely used neuropathic pain management agent, had an aOR of 3.16 (2.13–4.70) and another gabapentinoid, pregabalin, had an aOR of 2.21 (1.73–2.83). The loop-diuretic furosemide had a clear association with VTE incidence with an aOR of 2.05 (1.83–2.30).

**Table 5.** Ambulatory medications of the study population and associations with VTE.

	Controls (%)	Cases (%)	Overall (%)	OR^a^ (95%CI^b^)	aOR (95%CI)^c^
*n*	15,875	3404	19,279		
C01AA05 (digoxin)	451 (2.8)	101 (3.0)	552 (2.9)	0.79 (1.24–0.81)	0.72 (0.57–0.92)
C01DA02 (glyceryl trinitrate)	185 (1.2)	78 (2.3)	263 (1.4)	1.48 (2.56–1.75)	1.55 (1.16–2.01)
C01DA08 (isosorbide dinitrate)	429 (2.7)	168 (4.9)	597 (3.1)	1.43 (2.09–1.56)	1.35 (1.10–1.66)
C01DA14 (isosorbide mononitrate)	1037 (6.5)	368 (10.8)	1405 (7.3)	1.52 (1.98–1.53)	1.35 (1.17–1.56)
C02AC05 (moxonidine)	151 (1.0)	55 (1.6)	206 (1.1)	1.24 (2.31–1.42)	1.36 (0.98–1.90)
C03AA03 (hydrochlorothiazide)	181 (1.1)	39 (1.1)	220 (1.1)	0.70 (1.41–0.87)	0.88 (0.61–1.27)
C03CA01 (furosemide)	1518 (9.6)	718 (21.1)	2236 (11.6)	2.36 (2.91–2.16)	2.05 (1.83–2.30)
C03DA01 (spironolactone)	256 (1.6)	113 (3.3)	369 (1.9)	1.68 (2.65–1.72)	1.50 (1.18–1.91)
C03EA01 (hydrochlorothiazide and potassium-sparing agents)	500 (3.1)	94 (2.8)	594 (3.1)	0.70 (1.10–0.81)	0.85 (0.68–1.08)
C03EB01 (furosemide and potassium-sparing agents)	148 (0.9)	49 (1.4)	197 (1.0)	1.04 (2.04–1.22)	1.23 (0.87–1.74)
C07AA05 (propranolol)	118 (0.7)	35 (1.0)	153 (0.8)	0.96 (2.07–1.24)	1.29 (0.86–1.92)
C07AB02 (metoprolol)	1561 (9.8)	422 (12.4)	1983 (10.3)	1.13 (1.44–1.15)	1.06 (0.94–1.20)
C07AB03 (atenolol)	215 (1.4)	41 (1.2)	256 (1.3)	0.65 (1.27–0.82)	0.81 (0.56–1.16)
C07AB07 (bisoprolol)	2956 (18.6)	827 (24.3)	3783 (19.6)	1.29 (1.54–1.24)	1.14 (1.04–1.26)
C07AG02 (carvedilol)	221 (1.4)	58 (1.7)	279 (1.4)	0.90 (1.62–1.02)	1.03 (0.75–1.40)
C08CA01 (amlodipine)	1646 (10.4)	358 (10.5)	2004 (10.4)	0.90 (1.15–0.91)	0.88 (0.78–1.01)
C08CA02 (felodipine)	396 (2.5)	88 (2.6)	484 (2.5)	0.83 (1.34–0.92)	0.93 (0.83–1.19)
C08CA05 (nifedipine)	180 (1.1)	52 (1.5)	232 (1.2)	0.98 (1.84–1.31)	1.24 (0.89–1.72)
C08CA13 (lercanidipine)	549 (3.5)	100 (2.9)	649 (3.4)	0.68 (1.05–0.77)	0.78 (0.62–0.98)
C08DB01 (diltiazem)	144 (0.9)	48 (1.4)	192 (1.0)	1.15 (2.24–1.59)	1.35 (0.95–1.92)
C09AA02 (enalapril)	867 (5.5)	196 (5.8)	1063 (5.5)	0.88 (1.22–0.93)	0.91 (0.77–1.07)
C09AA04 (perindopril)	176 (1.1)	43 (1.3)	219 (1.1)	0.81 (1.59–0.99)	0.90 (0.63–1.30)
C09AA05 (ramipril)	1330 (8.4)	368 (10.8)	1698 (8.8)	1.20 (1.54–1.17)	1.23 (0.99–1.29)
C09BA02 (enalapril and diuretics)	445 (2.8)	87 (2.6)	532 (2.8)	0.73 (1.16–0.81)	0.82 (0.64–1.04)
C09CA01 (losartan)	735 (4.6)	182 (5.3)	917 (4.8)	1.00 (1.40–1.04)	1.00 (0.84–1.19)
C09CA03 (valsartan)	289 (1.8)	65 (1.9)	354 (1.8)	0.79 (1.37–0.93)	0.91 (0.69–1.22)
C09CA06 (candesartan)	597 (3.8)	146 (4.3)	743 (3.9)	0.95 (1.38–1.06)	1.03 (0.85–1.25)
C09CA07 (telmisartan)	176 (1.1)	35 (1.0)	211 (1.1)	0.65 (1.36–0.86)	0.97 (0.66–1.42)
C09DA01 (losartan and diuretics)	455 (2.9)	92 (2.7)	547 (2.8)	0.76 (1.20–0.81)	0.83 (0.65–1.05)
C09DA03 (valsartan and diuretics)	202 (1.3)	46 (1.4)	248 (1.3)	0.80 (1.52–1.01)	1.04 (0.74–1.46)
C09DA06 (candesartan and diuretics)	313 (2.0)	69 (2.0)	382 (2.0)	0.83 (1.40–0.98)	1.06 (0.81–1.39)
C10AA01 (simvastatin)	3116 (19.6)	636 (18.7)	3752 (19.5)	0.87 (1.05–0.82)	0.79 (0.72–0.88)
C10AA03 (pravastatin)	146 (0.9)	46 (1.4)	192 (1.0)	1.02 (2.01–1.36)	1.34 (0.94–1.90)
C10AA04 (fluvastatin)	228 (1.4)	41 (1.2)	269 (1.4)	0.59 (1.15–0.73)	0.70 (0.49–0.99)
C10AA05 (atorvastatin)	1186 (7.5)	256 (7.5)	1442 (7.5)	0.90 (1.20–0.91)	0.84 (0.72–0.97)
C10AA07 (rosuvastatin)	374 (2.4)	76 (2.2)	450 (2.3)	0.75 (1.25–0.85)	0.81 (0.63–1.06)
G03CA03 (estradiol)	500 (3.1)	77 (2.3)	577 (3.0)	0.56 (0.93–0.78)	0.78 (0.60–1.01)
H02AB04 (methylprednisolone)	31 (0.2)	47 (1.4)	78 (0.4)	4.70 (11.81–5.59)	5.37 (3.30–8.72)
H02AB06 (prednisolone)	276 (1.7)	251 (7.4)	527 (2.7)	3.80 (5.44–4.29)	4.05 (3.35–4.91)
H02AB07 (prednisone)	44 (0.3)	36 (1.1)	80 (0.4)	2.67 (6.62–4.26)	3.96 (2.4–6.44)
H03AA01 (levothyroxine sodium)	1238 (7.8)	364 (10.7)	1602 (8.3)	1.26 (1.63–1.34)	1.36 (1.17–1.59)
L04AX03 (methotrexate)	114 (0.7)	35 (1.0)	149 (0.8)	1.02 (2.19–1.41)	1.54 (1.03–2.29)
M04AA01 (allopurinol)	454 (2.9)	164 (4.8)	618 (3.2)	1.39 (2.01–1.34)	1.20 (0.99–1.47)
M05BA04 (alendronic acid)	201 (1.3)	54 (1.6)	255 (1.3)	0.94 (1.73–1.32)	1.35 (0.98–1.86)
M05BA07 (risedronic acid)	75 (0.5)	29 (0.9)	104 (0.5)	1.14 (2.73–1.84)	1.88 (1.19–2.96)
N03AE01 (clonazepam)	51 (0.3)	25 (0.7)	76 (0.4)	1.38 (3.65–2.02)	1.90 (1.13–3.22)
N03AG01 (valproic acid)	91 (0.6)	36 (1.1)	127 (0.7)	1.25 (2.74–1.45)	1.13 (0.73–1.72 (
N03AX12 (gabapentin)	66 (0.4)	49 (1.4)	115 (0.6)	2.44 (5.16–3.22)	3.16 (2.13–4.70)
N03AX16 (pregabalin)	220 (1.4)	118 (3.5)	338 (1.8)	2.04 (3.23–2.28)	2.21 (1.73–2.83)
N04BC05 (pramipexole)	116 (0.7)	42 (1.2)	158 (0.8)	1.20 (2.44–1.73)	1.86 (1.28–2.70)
N05AH03 (olanzapine)	84 (0.5)	52 (1.5)	136 (0.7)	2.13 (4.34–2.49)	1.94 (1.30–2.90)
N05AH04 (quetiapine)	200 (1.3)	98 (2.9)	298 (1.5)	1.77 (2.91–1.94)	1.72 (1.31–2.25)
N05AX08 (risperidone)	181 (1.1)	78 (2.3)	259 (1.3)	1.49 (2.57–1.81)	1.57 (1.19–2.14)
N05BA01 (diazepam)	98 (0.6)	36 (1.1)	134 (0.7)	1.22 (2.63–1.41)	1.30 (0.87–1.95)
N05BA04 (oxazepam)	276 (1.7)	136 (4.0)	412 (2.1)	1.94 (2.96–2.18)	1.80 (1.60–2.50)
N05BA06 (lorazepam)	86 (0.5)	36 (1.1)	122 (0.6)	1.34 (2.95–1.92)	1.80 (1.19–2.73)
N05CD07 (temazepam)	262 (1.7)	105 (3.1)	367 (1.9)	1.51 (2.43–1.71)	1.61 (1.25–2.06)
N05CF01 (zopiclone)	826 (5.2)	239 (7.0)	1065 (5.5)	1.18 (1.60–1.29)	1.22 (1.04–1.43)
N05CF02 (zolpidem)	194 (1.2)	43 (1.3)	237 (1.2)	0.74 (1.43–0.97)	0.97 (0.68–1.37)
N06AB04 (citalopram)	339 (2.1)	119 (3.5)	458 (2.4)	1.30 (2.01–1.52)	1.39 (1.11 (1.75)
N06AB10 (escitalopram)	196 (1.2)	61 (1.8)	257 (1.3)	1.10 (1.98–1.39)	1.38 (1.02–1.87)
N06AX11 (mirtazapine)	358 (2.3)	162 (4.8)	520 (2.7)	1.81 (2.66–2.11)	1.94 (1.58–2.38)
N06AX16 (venlafaxine)	99 (0.6)	49 (1.4)	148 (0.8)	1.66 (3.32–1.96)	1.94 (1.35–2.78)
N06DA02 (donepezil)	269 (1.7)	86 (2.5)	355 (1.8)	1.16 (1.92–1.58)	1.63 (1.26–2.12)
N06DA03 (rivastigmine)	155 (1.0)	46 (1.4)	201 (1.0)	0.94 (1.85–1.30)	1.33 (0.93–1.89)
N06DX01 (memantine)	272 (1.7)	106 (3.1)	378 (2.0)	1.42 (2.29–1.80)	1.84 (1.44–2.36)

^a^Odds ratio.

^b^Confidence interval.

^c^OR adjusted for all background variables (socioeconomic group, cancer diagnosis and type; prevalence of comorbidities, including diabetes, hypothyroidism, severe psychiatric disorders, chronic heart failure, coronary artery disease, hypertension, chronic arrhythmias, and inflammatory bowel disease).

Otherwise, many psychotropic agents from different drug classes had the strongest positive associations: Various benzodiazepines (oxazepam, clonazepam, lorazepam, temazepam), antipsychotics (olanzapine, quietapine, risperidone), dementia medications (memantine, donepezil), and an antidepressant agent venlafaxine all had aORs exceeding 1.5. Risedronic acid (osteoporosis agent) (1.88; 1.19–2.96) and a dopamine agonist pramipexole (1.86; 1.28–2.70) also exhibited a positive association with VTE incidence.

In contrast, the usage of statins was associated with a lower VTE incidence in our study (simvastatin 0.79; 0.72–0.88, fluvastatin 0.70; 0.49–0.99, atorvastatin 0.84; 0.77–0.97). Also, a dihydropyridine class calcium-channel blocker (DHB-CCB) and antihypertensive agent lercandipine had a significant protective association, with an aOR of 0.78 (0.62–0.97). Other DHB-CCBs, namely amlodipine and felodipine, also had aORs below 1, but the CIs were too wide to reach statistical significance. Digoxin, an agent that controls heart rate in atrial fibrillation, showed a protective association with an aOR of 0.72 (0.57–0.92).

### Predefined exposures and associations with VTE

As predefined, we conducted a second analysis of the study drugs with selected variables (Supplemental Table 2). Due to the infrequent exposure, we were unable to draw precise conclusions. However, a long-acting granulocyte colony-stimulating factor pegfilgrastim had a high aOR for VTE incidence (17.17; 4.80–6.12E1) with 21 exposures among the cases and three in the control group.

## Discussion

In our study, diabetes was an independent risk factor for VTE after adjustment for background variables. Additionally, inflammatory bowel disease and severe psychiatric disorders had significant associations with VTE in our diabetes-rich population, both of which are known risk factors in the general population.^1–5^ Among socioeconomic groups, students and pensioners had the strongest associations. The reasons for that remain speculative. Pensioners may also be less mobile than the occupational population, and their age exceeds 60 years, which has been shown the enhance the propensity for VTE.^1–5^ Diabetes medications that predispose to hypoglycemia (insulins and sulphonylureas) had stronger associations than metformin, which may reflect the reported prothrombotic effect of hypoglycemia.^19^ Our large, nested case-control study supports that the previously reported cancer type-related variation of thrombotic risk is also relevant in the Finnish diabetes-rich cohort. Many factors play a part in cancer-associated thrombosis, including the tumor cell biology, anatomic expansivity to the main vessels, therapy-related risks, and baseline characteristics. The most thrombotic cancer types in our study (pancreatic, stomach, ovarian, bronchus, and lung) are notorious for their extensive tissue factor expression, and in many cases, are diagnosed at the advanced stage, which also augments thrombogenicity.^20^

Our pharmaco-epidemiological data suggested some previously unrecognized risk medications (such as gabapentin, pregabalin, pramipexole, memantine, donepezil) for VTE. Indeed, both widely used gabapentinoids, gabapentin and pregabalin, had a significant positive association with VTE incidence. As these agents are mainly used for chronic pain syndromes (e.g., diabetic neuropathy), this association may be a surrogate for pronounced immobilization and other thrombotic lifestyle habits, as well as serious baseline comorbidities that were not adjusted for. The pharmacodynamic effects of gabapentinoids include binding to the von Willebrand factor (VWF)-containing calcium-channel alfa-2 delta subunits (found in the central nervous system, and skeletal and cardiac muscle cells), the potential hemostatic effects of which have not been studied.^35,36^ No data on the link between pramipexole and VTE is available, but the positive association in our study may reflect a thrombotic risk of Parkinson’s disease itself.^37^ However, pramipexole is also widely used among the non-Parkinson’s population.^38^ Memantine and donepezil, agents used for major cognitive impairment, have not previously been reported to carry thrombotic risk, but VTE has been reported as a major cause of death among people with advanced dementia, and immobilization and hypovolemia are overrepresented in this patient group.^39^

Our data highlight the already known prothrombotic effects of exogenous glucocorticoids,^22,24^ based on the increased levels of Factor VIII (FVIII) and VWF, leading to a hypercoagulable state.^22,24^ Corticosteroids are also used for several systemic diseases with thromboinflammatory properties. Granulocyte colony-stimulating factors promote neutrophilia and monocytosis, which both associate with thrombogenesis.^20,26^ These are usually used as supportive adjuncts in cancer care, but after adjustment for background variables, including cancers, pegfilgrastim still presented with a high aOR for VTE incidence, reflecting independent prothrombotic properties. Another positive association with risedronic acid may reflect the already reported thrombotic risk among osteoporosis patients that did not depend on whether they received pharmacological treatment.^40^ Furosemide continued to have a significant association with VTE incidence after adjustment for chronic heart failure. It is also widely used for non-cardiogenic edema, and the positive association of furosemide may reflect baseline non-adjusted thrombogenic conditions such dehydration, nephrotic syndrome, or chronic venous insufficiency.^1–5^

Severe psychiatric disorder as a baseline condition was associated with an increased risk of VTE. In accordance with this, psychotropic agents from different drug classes had significant positive associations with VTE. Exposure to antipsychotics and its relationship to VTE is an early observation, but the mechanisms and causation are unclear and complex. Sedation, obesity, and immobilization may play a central part, and drug-induced hyperprolactinemia is reported to enhance coagulation and possibly also platelet aggregation.^22,27,41,42^ In our study, olanzapine, quietapine, and risperidone were all associated with VTE incidence. In addition to antipsychotics, several benzodiazepines had a positive, previously reported association.^43^ Immobilization and sedation may be potential contributors to the thrombosis risk. All in all, thrombotic risk among patients with severe psychiatric disorders and psychotropic medications is important in clinical practice, and the threshold of adequate diagnostics should be low in cases of potential thrombotic symptoms. Considering the mean age of our cohort, psychotropic medications and benzodiazepines may also have been used to control dementia-related symptoms, which partly may explain the positive associations with VTE.^38^

With respect to likely protective medications, our data align with the earlier positive results of statin being associated with lower VTE incidence.^28,29^ Fluvastatin and simvastatin had the most significant protective associations. In diabetes population, lipophilic statin usage has been previously associated with marginally lower risk of VTE without statistical significance.^44^ In addition, DHB-CCB lercanidipine was also associated with a significantly lower risk. Other DHB-CCBs, namely amlodipine and felodipine, also exerted non-significant protection, which suggests a potential group effect. Calcium ions are the major regulators of coagulation cascade, and previous evidence suggests that CCBs may have a protective association with stent thrombosis in coronary artery disease,^45^ and vice versa, may potentiate the risk of GI bleeding in hypertensive patients.^46^ However, the relationship between DHP CCBs and VTE has not been previously reported.

Our study has certain limitations. Prevalence of diabetes was high, and the mean age of the cohort was 65, which considerably limits the generalizability of the results. In our study, the definition of diabetes was based on the reimbursement and redeeming of at least one insulin product or oral antidiabetic agent. It is possible that some patients with early diabetes were treated only with life-style interventions, not with medication. However, as behavioral modification is challenging and may just delay the start of drug treatment, the Finnish Current Care guideline (original version published in 2007 and thereafter updated several times) recommends that metformin should be initiated concomitantly with life-style interventions unless contraindicated. These guidelines have been extensively implemented in primary care,^47^ and therefore it is likely that those clinically diagnosed with diabetes were included in the study. We included the diagnoses I26, I63.6 and I80 due to their common real-world national use in clinical practice, but it is possible that some DVT diagnoses are not included in the I80 category. However, the large mortality study of PE, Finnish data included, reveals that among fatal DVT ICD codes, I80 predominates while I82 diagnoses are rare.^48^

In some of the subgroups analyzed (especially regarding some cancer types and ambulatory medications), the sample sizes were small, compromising the statistical certainty of the results. For example, 95% CIs for the most prothrombotic cancers (pancreatic, stomach, ovarian, and bronchus and lung) were quite wide. All thrombogenicity-related baseline conditions (e.g., body mass index, autoimmune conditions, dyslipidemia, chronic kidney disease, hereditary and acquired thrombophilia)^1–5^ could not be adjusted because of unavailable data. Additionally, information about major transient risk factors for VTE, like major surgery, infections or immobilization to name a few, could not be acquired in the frames of this study. We were unable to obtain data on antithrombotic medications, but we did adjust for chronic arrythmias and coronary artery disease, as many people with anticoagulation or antiplatelet medication have these diagnoses. We also excluded people with previous VTE events, and thus possible ongoing anticoagulation. As said, we excluded people with a history of VTE requiring hospital or tertiary care to make the associations less confounded by the high-risk patients with recurrent VTE. However, some less severe VTE events are diagnosed and treated by primary health care providers and could not be excluded. Positive associations between drugs and VTE incidence may sometimes instead reflect the thrombogenicity of the possible unadjusted baseline condition rather than the drug itself. Further research is required to establish the causation of the specific drugs and thrombosis.

## Conclusion

VTE risk varied considerably according to the tumor type, and pancreatic, stomach, ovarian, bronchus, and lung cancers are associated with the highest VTE risk among a patient cohort of which 50% had diabetes. The thrombogenicity among people with major psychiatric disorders and/or psychotropic medication were highlighted in our cohort.

As novel observations, we found some previously unrecognized potential risk medications for VTE, but also a possible protective association of lercandipine and VTE, these data will require confirmation in other patient cohorts.

## Supplemental Material


Supplemental Material - A pharmacoepidemiological nested case-control study of risk factors for venous thromboembolism with the focus on diabetes, cancer, socioeconomic group, medications, and comorbidities
Supplemental Material for A pharmacoepidemiological nested case-control study of risk factors for venous thromboembolism with the focus on diabetes, cancer, socioeconomic group, medications, and comorbidities by Lasse Myllylahti, Leo Niskanen, Riitta Lassila and Jari Haukka in Journal of Diabetes & Vascular Disease Research.

## Data Availability

We are open to data sharing upon reasonable request.
