# Supplementary material for: A pharmacoepidemiological nested case-control study of risk factors for venous thromboembolism with the focus on diabetes, cancer, socioeconomic group, medications, and comorbidities
Source: Diab Vasc Dis Res. 2024 Jun 21;21(3):14791641241236894. doi: 10.1177/14791641241236894 (PMC11193353; doi:10.1177/14791641241236894)
Supplement: Supplemental Material - A pharmacoepidemiological nested case-control study of risk factors for venous thromboembolism with the focus on diabetes, cancer, socioeconomic group, medications, and comorbidities [file sj-pdf-1-dvr-10.1177_14791641241236894.pdf]

Supplementary table 1: Drug classes	ATC*
Antiobesity preparations (excluding diet products)	A08A
Drugs used in diabetes	A10
Cardiovascular system	C
Sex hormones and modulators of the genital system	G03
Pituitary and hypothalamic hormones and analoques	H01
Corticosteroids for systemic use	H02
Thyroid therapy	H03
Antineoplastic agents	L01
Endocrine therapy	L02
Immunostimulants	L03
Immunosuppressants	L04
Drugs for treatment of bone diseases	M05
Antiepileptics	N03
Anti-Parkinson drugs	N04
Psycholeptics	N05
Psychoanaleptics	N06
Other nervous system drugs	N07

* Anatomical therapeutic chemical code

Supplementary table 2: predefined exposures	ATC*	Controls(%)	Cases (%)	Overall (%)
n		15875	3404	19279
Testosterone	G03BA03	39(0.2)	14(0.4)	53(0.3)
Estradiol	G03CA03	500(3.1)	77(2.3)	577(3.0)
Estriol	G03CA04	7(0.0)	0(0.0)	7(0.0)
Tibolone	G03CX01	22(0.1)	0(0.0)	22(0.1)
Norethisterone and estrogen	G03FA01	62(0.4)	6(0.2)	68(0.4)
Medroxyprogesterone and estrogen	G03FA12	18(0.1)	3(0.1)	21(0.1)
Dydrogestrone and estrogen	G03FA14	14(0.1)	2(0.1)	16(0.1)
Drospirenone and estrogen	G03FA17	8(0.1)	2(0.1)	10(0.1)
Norethisterone and estrogen	G03FB05	14(0.1)	1(0.0)	15(0.1)
Medroxyprogesteroene and estrogen	G03FB06	3(0.0)	0(0.0)	3(0.0)
Dydrogestrone and estrogen	G03FB08	8(0.1)	1(0.0)	9(0.0)
Levonorgestrel and estrogen	G03FB09	1(0.0)	0(0.0)	1(0.0)
Cyproterone and estrogen	G03HB01	1(0.0)	0(0.0)	1(0.0)
Tamoxifen	L02BA01	21(0.1)	7(0.2)	28(0.1)
Filgrastim	L03AA02	7(0.0)	3(0.1)	10(0.1)
Pegfilgrastim	L03AA13	3(0.0)	21(0.6)	24(0.1)
Lenalidomide	L04AX04	6(0.0)	2(0.1)	8(0.0)
Clozapine	N05AH02	23(0.1)	10(0.3)	33(0.2)

Simvastatin	C10AA01	3116(19.6)	636(18.7)	3752(19.5)
Lovastatin	C10AA02	91(0.6)	32(0.9)	123(0.6)
Pravastatin	C10AA03	146(0.9)	46(1.4)	192(1.0)
Fluvastatin	C10AA04	228(1.4)	41(1.2)	269(1.4)
Atorvastatin	C10AA05	1186(7.5)	256(7.5)	1442(7.5)
Rosuvastatin	C10AA07	374(2.4)	76(2.2)	450(2.3)
Simvastatin and ezetimibe	C10BA02	4(0.0)	1(0.0)	5(0.0)

* Anatomical therapeutic chemical code

3
